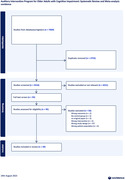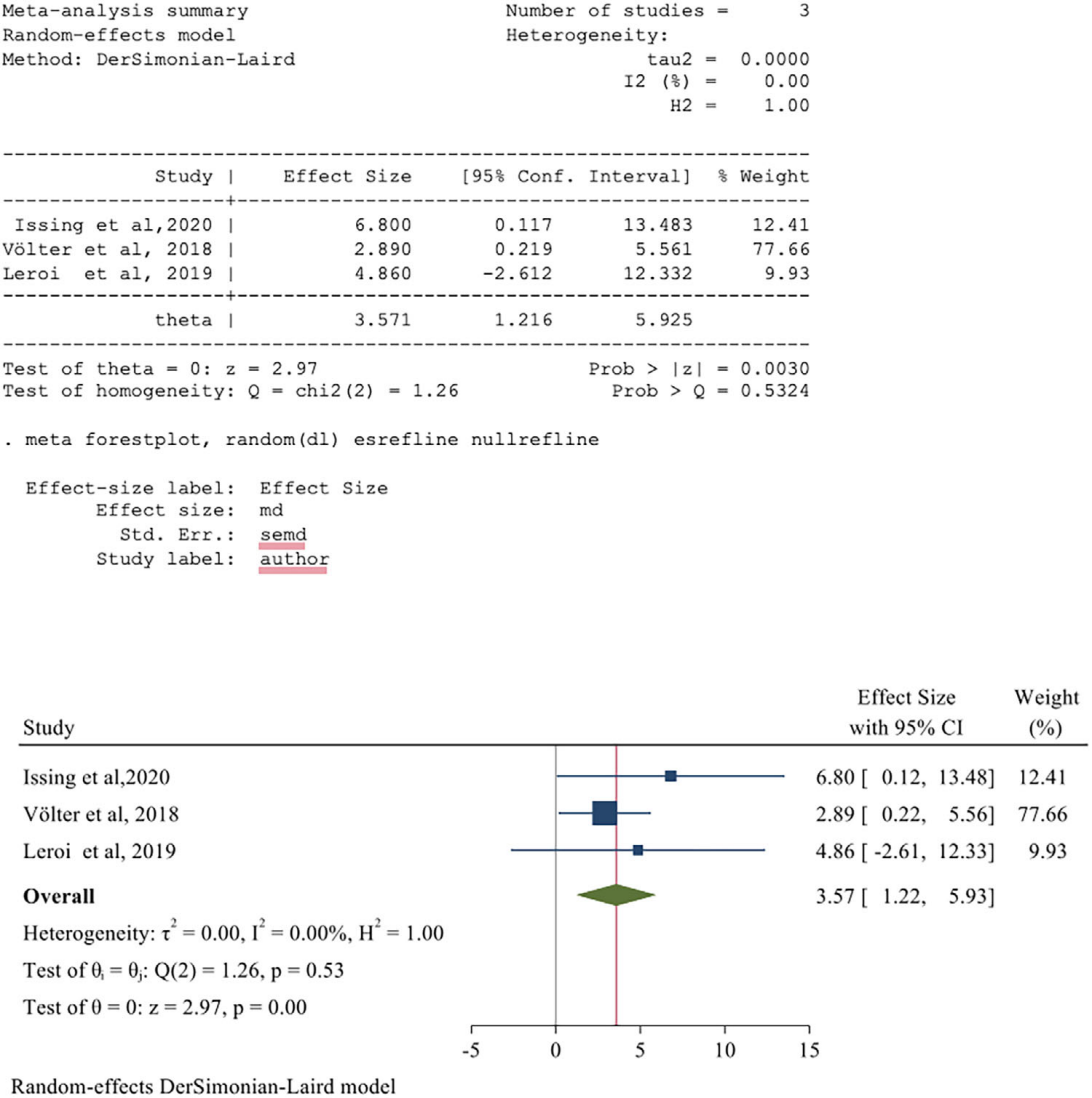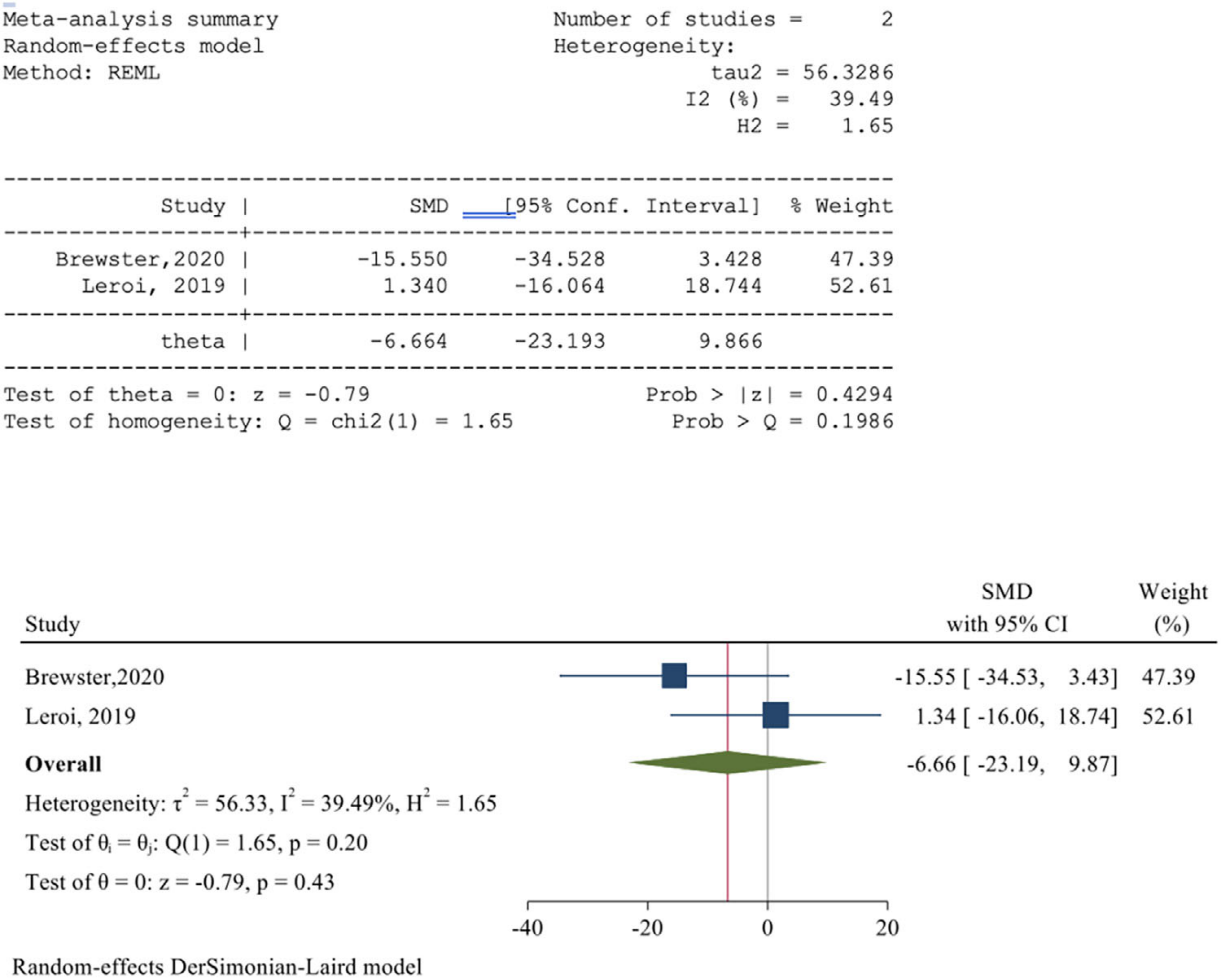# Auditory Intervention Program for Older Adults with Cognitive Impairment: Systematic Review and Metaanalysis

**DOI:** 10.1002/alz.093674

**Published:** 2025-01-09

**Authors:** Teeraya Piyajarawong, Patcharaorn Limkitisupasin, Pornnapat Manum, Nattawan Utoomprurkporn

**Affiliations:** ^1^ Chulalongkorn University, BKK, Bangkok Thailand; ^2^ Faculty of Medicine, Chulalongkorn University, Bangkok Thailand; ^3^ Chulalongkorn University, Bangkok, Bangkok Thailand

## Abstract

**Background:**

The elderly population is particularly susceptible to the development of age‐related hearing loss and cognitive impairment. The gradual decline in auditory perception often goes unnoticed, leading to a detrimental impact on their quality of life, mental well‐being, and overall communication skills. Extensive research indicates that prolonged social isolation can exacerbate cognitive decline. Fortunately, hearing intervention has the potential to prevent this devastating consequence.

**Method:**

Three independent reviewers meticulously reviewed the selected articles. The Covidence was utilized to identify and eliminate any duplicate publications. In cases of disagreement, a fourth reviewer was consulted for adjudication. For inclusion criteria, the studies had to be in the English language and conducted on a population aged 50 years or older who were diagnosed with cognitive impairment. Animal studies and those lacking control groups (either healthy controls or pre‐ and post‐intervention tests) were excluded. We utilized the National Institute of Health (NIH) criteria for evaluating the quality of studies.

**Result:**

Out of the initial 7,069 search results, we selected 39 articles for inclusion in this study. Among these, the majority comprised randomized trials (10 out of 39) and pre‐ and post‐intervention tests (10 out of 39). The hearing interventions were categorized into four distinct groups: hearing aids, cochlear implants, medication, and sound therapy. It was observed that medication showed the least efficacy while hearing aids and cochlear implants demonstrated the most significant improvements. Following the prescription of hearing aids and cochlear implants, patients experienced enhanced quality of life, despite significant improvement not only in cognition but also in psychological and social well‐being. Additionally, sound therapy exhibited promising results in terms of stress relief and improvement in instrumental and lyric recognition among cognitively impaired patients.

**Conclusion:**

Our findings indicate that hearing aids, cochlear implants, and sound therapy significantly enhance psychological and social health in individuals with cognitive impairment or dementia. Furthermore, there is a statistically significant improvement in quality of life with appropriate hearing interventions. However, we observed no substantial improvement in cognitive status as assessed by the Montreal Cognitive Assessment (MoCA) and the Mini‐Mental State Examination (MMSE), although there is a suggestive trend towards cognitive enhancement.